# A real-world study of Afatinib plus ramucirumab in treatment-naïve, EGFR-mutated, non–small cell lung cancer

**DOI:** 10.1186/s12885-023-10909-z

**Published:** 2023-05-08

**Authors:** Chun-Yao Huang, Hui-Li Huang, Chou-Chin Lan, Yi-Chih Huang, Yao-Kuang Wu

**Affiliations:** Division of Pulmonary Medicine, Taipei Tzu Chi Hospital, Buddhist Tzu Chi Medical Foundation, No. 289, Jianguo Rd, Xindian District, 231 New Taipei City, Taiwan

**Keywords:** Afatinib, Ramucirumab, Non–small cell lung cancer, *EFGR* mutation

## Abstract

**Background:**

Recent reports suggested combining ramucirumab with epidermal growth factor receptor (EGFR)-tyrosine kinase inhibitors (TKIs) to overcome EGFR resistance in non–small cell lung cancer (NSCLC). Nonetheless, evidence supporting the activity of afatinib and ramucirumab is lacking. This study investigated the survival benefits and safety profile of afatinib plus ramucirumab in patients with treatment-naïve, EGFR-mutated, metastatic NSCLC.

**Materials and methods:**

The medical records of patients with EGFR-mutated NSCLC were retrospectively retrieved. Patients who received first-line sequential afatinib followed by ramucirumab and the first-line combination of afatinib plus ramucirumab were included. The Kaplan-Meier was used to estimate the progression-free survival (PFS) of all included patients, patients on sequential afatinib followed by ramucirumab (PFS1), and patients on the up-front combination of afatinib and ramucirumab (PFS2).

**Results:**

Thirty-three patients were included (25 women; median age: 63 [45–82] years). The median follow-up of the included patients was 17 months (range 6–89 months). the median PFS for the whole cohort was 71 months (95% CI 67.2–74.8) with eight events during the follow-up. The median PFS1 and PFS2 were 71 months (95 CI not defined) and 26 months (95% CI 18.6–33.4), respectively. In terms of OS, the median OS for all patients and patients on sequential treatment was not defined, while the median OS for patients on upfront combination was 30 months (95% CI 20.9–39.1). There was no significant association between EGFR mutation type and PFS1 or PFS2.

**Conclusions:**

Afatinib plus ramucirumab could improve the PFS of patients with EGFR-positive NSCLC at a predictable safety profile. Our data also suggest a survival benefit of adding ramucirumab to afatinib in patients with uncommon mutations, which should be investigated further.

## Introduction

According to GLOBOCAN 2020, lung cancer is the second most common cancer and the leading cause of cancer-related death worldwide [1]. Non–small cell lung cancer (NSCLC) accounts for nearly 85% of primary lung cancer cases. NSCLC ‘tends to metastasize at early stages so that up to 35% of NSCLC patients present with *de novo* brain metastasis [2]. Brain metastasis is the major cause of morbidity and mortality in NSCLC patients. Treatment of brain metastasis is a great challenge as the blood-brain barrier (BBB) prevents the entry of most chemotherapeutics into the brain.

Recent advances in molecular oncology have improved our understanding of genetic and epigenetic regulations of NSCLC tumorigenesis and cell survival [3]. Epidermal growth factor receptor (EGFR) is a transmembrane glycoprotein receptor with an intracellular tyrosine kinase component implicated in cell proliferation and survival regulations. The current evidence shows the significant involvement of EGFR overexpression in developing several malignancies, including NSCLC [4]. The frequency of EGFR mutations in NSCLC cases shows substantial ethnic and geographical disparity, with the highest prevalence observed among patients from the Asia-Pacific region (range 20–76%). In Taiwan, the frequency of EGFR mutations was estimated to be as high as 76% amongst NSCLC cases [5]. Both deletion within exon 19 (ex19del) and leucine to arginine substitution mutation in exon 21 (Leu858Arg) account for nearly 90% of EGFR mutations in NSCLC patients [6]. These activating EGFR mutations are responsive to small-molecule EGFR tyrosine kinase inhibitors (TKIs) [6, 7].

Clinical trials and real-world evidence have established the efficacy of afatinib, a second-generation EGFR-TKI, as the first-line treatment of choice for EGFR mut^+^ mNSCLC patients [8]. Afatinib is a second-generation EGFR-TKI that irreversibly blocks the ErbB family of protein-tyrosine kinases. Clinical evidence demonstrated that afatinib can pass the BBB [9]. Two landmark randomized controlled trials (RCTs) (LUX-Lung 3 and Lux-Lung 6) demonstrated a significant improvement in objective response rate (ORR) and progression-free survival (PFS) with afatinib compared with platinum-based chemotherapy as first-line treatment in EGFR-mutated metastatic NSCLC [10–12]. In Taiwan, afatinib is reimbursed by the National Health Insurance (NHI) as a first-line option for EGFR-mutated metastatic NSCLC [13]. Unfortunately, data from clinical trials showed that most patients experience tumor progression after 10–14 months [14]. A combination therapy with other targeted agents is a viable choice to reduce the rates of resistance to EGFR-TKI. In 2009, Naumov et al. demonstrated that the dual inhibition of EGFR and VEGF abrogates the *EGFR resistance* in NSCLC models. The authors concluded that *EGFR resistance* is a VEGF-mediated process, and combined blockade of the VEGFR and EGFR pathways can overcome EGFR resistance [15]. Ramucirumab is a fully human IgG1 monoclonal antibody that specifically binds to the extracellular domain of vascular endothelial growth factor receptor 2 (VEGFR-2) with high affinity, preventing the binding of VEGF ligands and inhibiting receptor activation [16]. The CNS activity of ramucirumab has been demonstrated in clinical studies [17–20]. The groundbreaking phase III double-blind RELAY trial demonstrated that adding ramucirumab to erlotinib improved PFS (19.4 vs.12.4 months) in treatment-naïve EGFR-mutated metastatic NSCLC [21]. A phase Ib trial, which recruited Japanese patients with advanced EGFR-mutated metastatic NSCLC, showed a tolerable safety profile of afatinib plus ramucirumab and a median PFS of 9.2 months [22]. Still, the current literature is scarce regarding the benefits of adding ramucirumab to first-line EGFR TKIs, such as afatinib, in patients with treatment-naïve NSCLC. Therefore, we conducted this retrospective study to investigate the survival benefits and safety profile of afatinib plus ramucirumab in patients with treatment-naïve, EGFR-mutated metastatic, NSCLC.

## Materials and methods

The research was approved by the institutional review board of the Tzu Chi Hospital, Taiwan. All procedures were in line with the latest version of the Declaration of Helsinki, and the report was prepared according to the STROBE statement [23].

### Eligibility criteria and data collection

For this retrospective study, the medical records of patients with treatment-naïve stage IV NSCLC and laboratory-confirmed EGFR mutation (ex19del, L858R, or rare mutations, including exon 20 insertions, S768I, or L861Q), whether they had *de novo* brain metastases per contrast-enhanced magnetic resonance imaging (MRI) or not, were retrieved. This study included patients who initiated treatment with either sequential afatinib (30 mg/d) followed by ramucirumab (10 mg/kg) or front-line combination of afatinib (30 mg/d) plus ramucirumab (10 mg/kg). Data of 33 patients who were treated between March 1, 2016, and April 30, 2022, were retrieved.

Data regarding the demographic characteristics, tumor stage, EGFR mutation type, treatment regimens, response rate, follow-up duration, progression status, and adverse events were collected. The 7th and 8th editions of TNM staging system were used to classify patients into stage Iva or Ivb. The Response Evaluation Criteria in Solid Tumors (RECIST) version 1.1 was used to evaluate treatment response, while the adverse events were classified according to the Common Terminology Criteria for Adverse Events (CTCAE) version 5.0.

The primary endpoint was to investigate the PFS of the combination treatment. Other endpoints included the PFS according to EGFR mutation, ORR (defined as complete response rate plus partial response rate), and the incidence of treatment-related adverse events.

### Statistical analysis

The results were analyzed using SPSS version 28 software for Windows (IBM Corp. Armonk, NY, USA). Descriptive statistics were employed according to data type. The Kaplan-Meier was used to estimate the PFS of all included patients, patients on sequential afatinib followed by ramucirumab (PFS1), and patients on up-front combination of afatinib and ramucirumab (PFS2). The PFS was calculated as the time from treatment initiation to progression or death from any cause. A log-rank test was used to compare the PFS according to EGFR mutation status. The chi-square test, Yate’s correction when needed, compared the incidence of adverse events between patients who initiated afatinib only and patients who initiated afatinib plus ramucirumab. The results were considered significant when two-tailed P < 0.05.

## Results

### Patients’ clinical characteristics

Thirty-three patients were included, with female predominance (n = 25; 75.8%) and a median age of 63 (range 45–82) years. Only two patients (6.1%) were smoker. Twenty-seven patients (81.8%) had stage IVB NSCLC. All patients had an Eastern Cooperative Oncology Group performance status (ECOG PS) of 0 or 1. The distribution of the EGFR mutations was as follows: ex19del (42.4%), L858R (51.5%), G719 × (3%), and L861Q (3%). Fifteen patients had brain metastasis, 60% of them had multiple sites of brain metastasis. One-third of the patients with brain metastasis were symptomatic. Overall, out of the 15 patients, 13 (86.7%) received local radiotherapy for brain metastasis before initiating Afatinib or during the course of treatment.

In this study, 11 patients administered the sequential regimen (afatinib followed by ramucirumab) and 22 patients received upfront combination therapy (afatinib plus ramucirumab). In patients who received sequential regimen, the median duration between starting afatinib and starting ramucirumab was 35 months (range 9–43 months), Table 1.

**Table 1:** Characteristics of the patients

Characteristics	Patients (n = 33)
Median age (years), n (%)	63 (45–82)
< 70	26 (78.8%)
≥ 70	7 (21.2%)
Sex, n (%)	
Male	8 (24.2%)
Female	25 (75.8%)
Smoking status, n (%)	
Non-smoker	31 (93.9%)
Smoker	2 (6.1%)
Clinical stage ^a^, n (%)	
IVA	6 (18.2%)
IVB	27 (81.8%)
ECOG PS, n (%)	
0	0 (0.0%)
1	33 (100.0%)
EGFR mutation, n (%)	
Del 19	14 (42.4%)
L858R	17 (51.5%)
Other ^a^	2 (6.1%)
Brain metastasis, n (%)	15 (46.5%)
No. Brain metastasis, n (%)	n = 15
Single	6 (40%)
Multiple	9 (60%)
Location of Brain metastasis, n (%)	n = 15
Cerebellum	1 (6.7%)
Cerebellum, Cerebrum	5 (33.3%)
Cerebrum	9 (60%)
Oligo-metastatic, n (%)	n = 15
Single	5 (33.3%)
Multiple	2 (13.3%)
Leptomeningeal metastasis, n (%)	2 (13.3%)
Symptomatic brain metastasis, n (%)	5 (33.3%)
Treatment of brain metastasis, n (%)	n = 15
Surgery	0
Radiotherapy	13 (86.7%)
Treatment, n (%)	
Sequential afatinib followed by ramucirumab	11 (33.3%)
Upfront combination	22 (66.7%)
Response, n (%)	
CR and PR	33 (100.00%)
SD	0 (0.00%)
Progressive disease, n (%)	
Yes	5 (15.2%)
No	28 (84.8%)

Abbreviation: CR, complete response; ECOG PS, Eastern Cooperative Oncology Group performance status; PR, partial response; SD: stable disease

^a^ Based on American Joint Committee on Cancer (7th and 8th editions)

^b^ Rare EGFR mutations, including G719X and L861Q

### Progression-free survival and overall survival

**Fig. 1:**
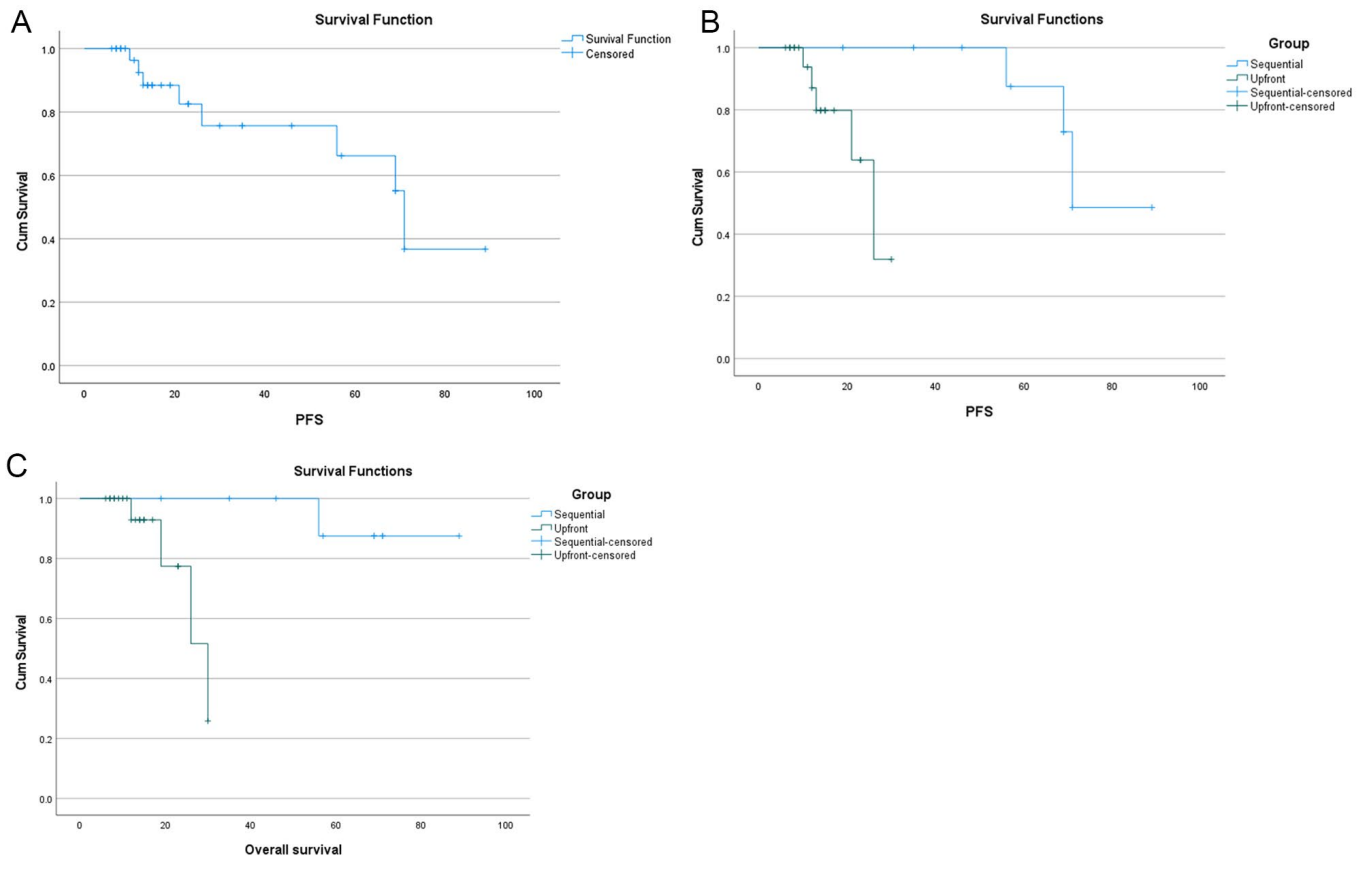
(**A**) Kaplan-Meier estimates of progression-free survival (mPFS = 71 mon) of all patients who received either upfront combination or sequential treatment with afatinib and ramucirumab (n = 33 patients); (**B**) Kaplan-Meier estimates of progression-free survival in patients received sequential (blue; mPFS 71 mon; n = 11) and upfront combination therapy (green; mPFS 30 mon; n = 22). PFS, progression-free survival; (**C**) Kaplan-Meier estimates of overall survival in patients received sequential (blue; mOS not defined mon; n = 11) and upfront combination therapy (green; mOS 30 mon; n = 22)

**Fig. 2:**
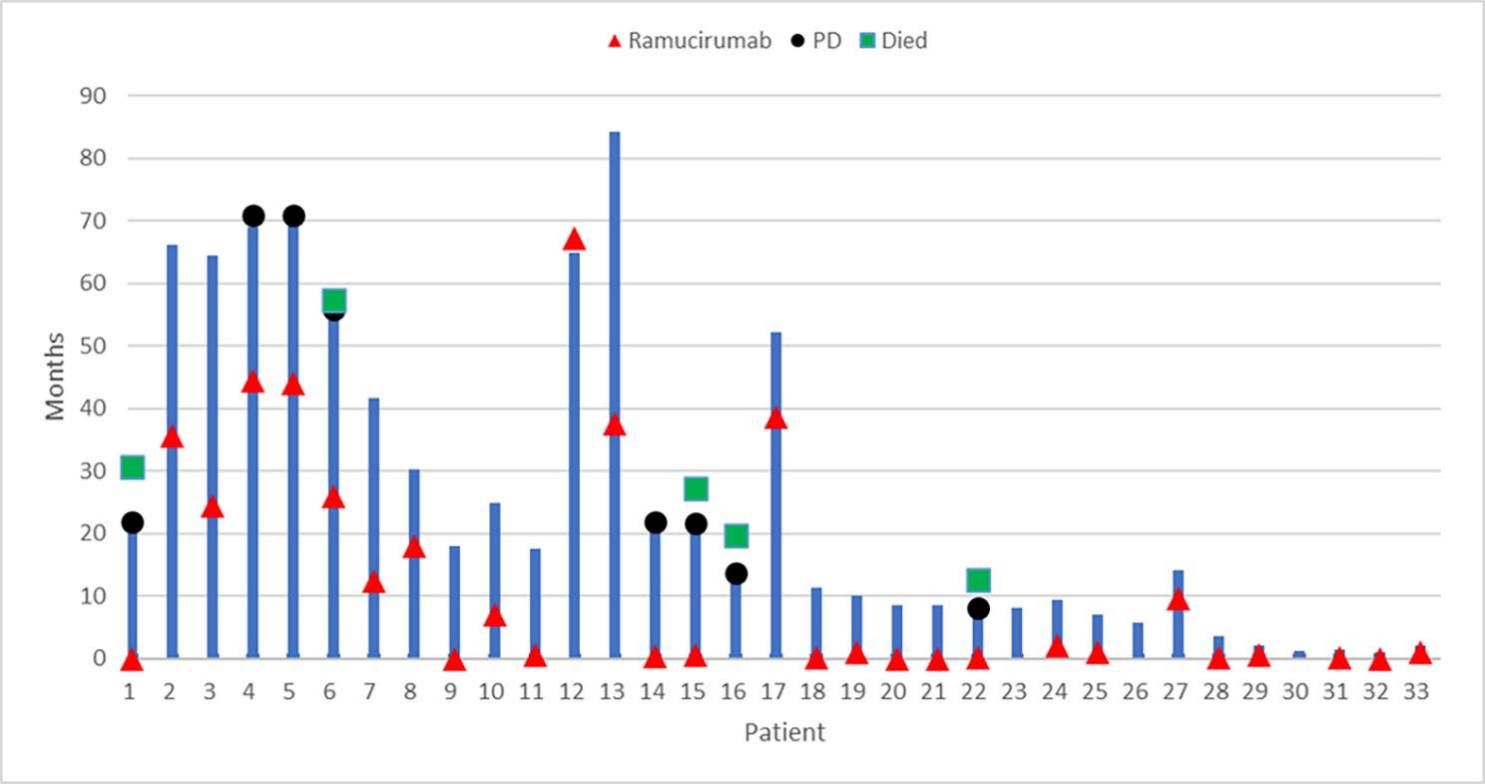
Swimmer plot of the 33 patients. Kaplan-Meier estimates of progression-free survival of patients with L858R and ex19del EGFR mutations (**A**) patients starting sequential regimen; (**B**) patients starting up-front combination

The median follow-up of the included patients was 17 months (range 6–89 months). The PFS was determined for all patients who received either upfront combination or sequential treatment with afatinib and ramucirumab (n = 33 patients); the median PFS for the whole cohort was 71 months (95% CI 67.2–74.8) with eight events during the follow-up (Fig. 1A). The median PFS1 and PFS2 were 71 months (95 CI not defined) and 26 months (95% CI 18.6–33.4), respectively (Fig. 1B). In terms of OS, the median OS for all patients and patients on sequential treatment was not defined, while the median OS for patients on upfront combination was 30 months (95% CI 20.9–39.1), Fig. 1C. Figure 2 shows the swimmer plot of the 33 patients.

### Association between EGFR mutations and treatment outcomes

**Fig. 3:**
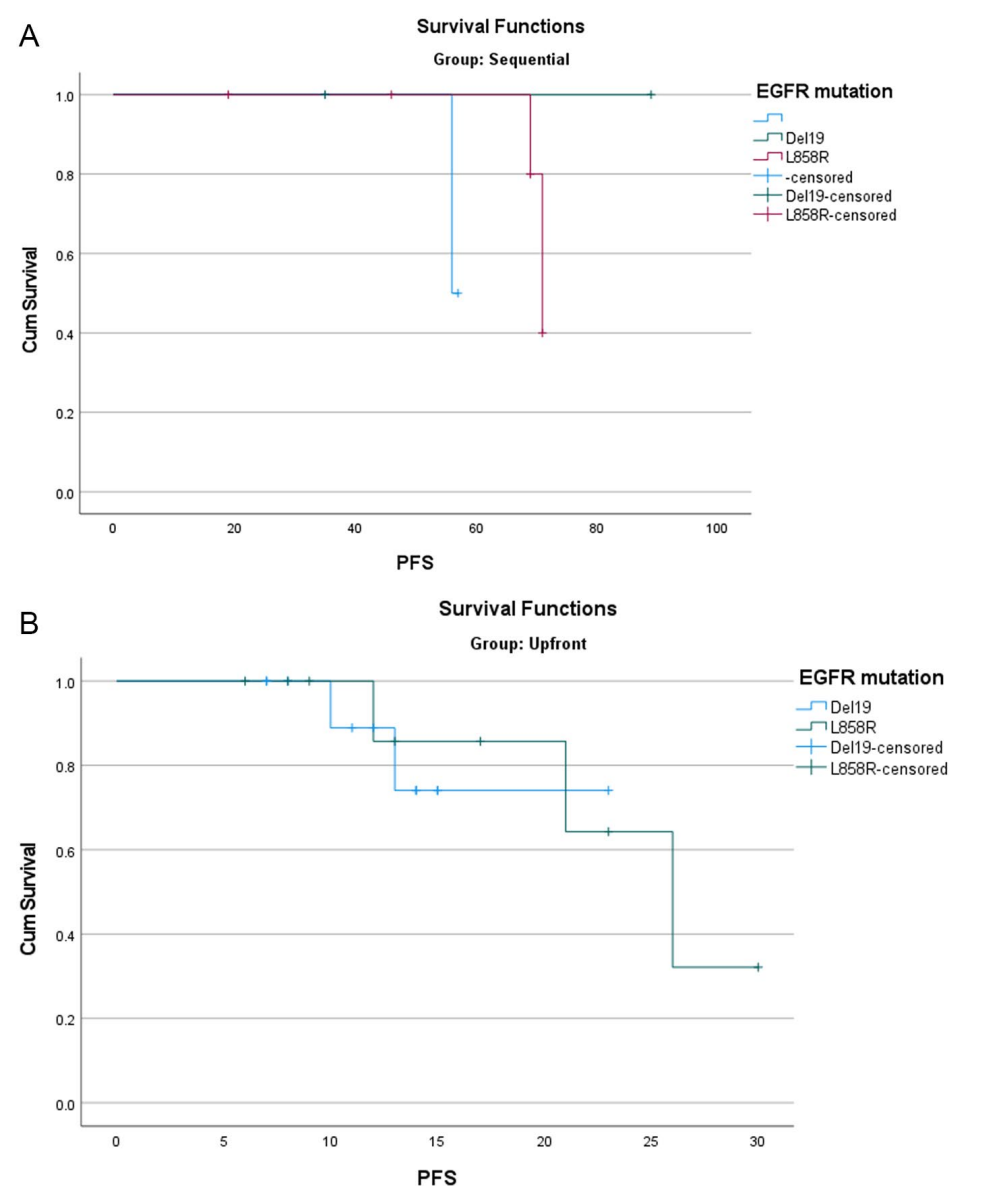
Kaplan-Meier estimates of progression-free survival of patients with L858R and ex19del EGFR mutations (**A**) patients starting sequential regimen; (**B**) patients starting up-front combination

There was no significant association between EGFR mutation type and PFS1 (*P* = 0.171; Fig. 3A). The median PFS1 of patients with L858R and ex19del was undefined. Likewise, there was no significant association between EGFR mutation type and PFS2 (P = 0.803; Fig. 3B). The median PFS2 of patients with L858R was 26 months (95% CI 18.4–33.6), while the estimate was not defined for patients with ex19del mutation.

In the present study, two patients presented with uncommon EGFR mutations. The first patient was a female aged 77 years who presented with stage IVA adenocarcinoma and metastatic pleural effusion. She had L861Q positive mutation. She initially started on 30 mg afatinib and then received 10 mg/kg ramucirumab two years later. She has an ongoing response with a notable PFS of > 56 months. The second patient was a 65 years old female with a stage IVB disease who started 30 mg afatinib in 2018. Three years later, she received 10 mg/kg ramucirumab. The patient had G719X mutation and showed an ongoing PFS of > 52 months.

### Adverse events

The adverse events caused by each type of treatment regimens were analyzed. Despite being frequent among patients, the presentation of diarrhea (*P* = 0.602) and paronychia (*P* = 0.801) had no significant difference between patients who were first treated with sequential regiment and patients treated with up-front combination (Table 2).

**Table 2:** Presence or absence of adverse events

Adverse Events (N = 33)	Sequential (n = 11)	Up-front combination (n = 22)	P-value
Diarrhea	8 (72.7%)	14 (63.6%)	0.602
Dermatitis	11 (100%)	22 (100%)	--
Paronychia	7 (63.6%)	13 (59.1%)	0.801
Nausea & Vomiting	1 (9.1%)	0 (0%)	0.151
Mucositis	0 (0%)	0 (0%)	--
Hepatitis	0 (0%)	0 (0%)	--
Hemorrhagic events	0 (0%)	0 (0%)	--
Renal dysfunction	0 (0%)	0 (0%)	--

## Discussion

Ramucirumab represents a viable option to overcome *EGFR resistance* in patients with EGFR-mutated metastatic NSCLC. The phase III RELAY trial found that ramucirumab addition to erlotinib improves disease control in patients with EGFR-mutated metastatic NSCLC [21]. In addition, the safety profile of ramucirumab plus erlotinib was demonstrated in several trials [21, 22]. However, studies are yet to determine the clinical efficacy and safety profile of upfront ramucirumab plus EGFR-TKI combination in NSCLC patients. The present study found that the median PFS of the EGFR-mutated patients on ramucirumab plus afatinib, whether the combination was initiated concurrently or sequentially, was 71 months (95% CI not defined). Besides, the median PFS for patients who initiated sequential treatment and upfront combination were 71 months (95% CI not defined) and 26 months (95% CI 18.6–33.4), respectively. Furthermore, no significant difference in PFS was observed among patients with different EGFR mutations. To our knowledge, this is the first study that outlines the efficacy of afatinib plus ramucirumab in treatment-naïve patients with EGFR mutation. Of note, the median PFS observed in our cohort was notably longer than the median PFS reported in pivotal clinical trials and observational studies. In the LUX-Lung 3, 6, and 7 trials, EGFR-mutated patients with brain metastasis on first-line afatinib had a median PFS ranging from 7.2 to 8.2 months [11, 12, 24]. Similar figures were reported in real-world studies (median PFS = 8.2 months) [25]. Thus, our data suggest that ramucirumab improves the PFS of first-line EGFR-TKI. These run in line with previous reports showing an improvement in PFS amongst patients receiving first-generation EGFR-TKIs plus ramucirumab [21]. Further experimental evidence is needed to elucidate the potential synergistic mechanisms of action of the combination therapy.

Uncommon EGFR mutations can present in 10% of the NSCLC patients and show variable responses to EGFR-TKIs [26]. The survival benefits of afatinib in patients with uncommon EGFR mutations are well established, and it is currently approved for patients with any EGFR mutation. According to a subgroup analysis from LUX-Lung 2, 3, and 6 trials, patients harboring G719X and L861Q mutations had a median PFS of 13.8 and 8.2 months, respectively [27]. Two patients presented with uncommon EGFR mutations in the present retrospective chart review. The patient with L861Q mutation had a notable PFS of > 56 months, while the patient with G719X mutation showed an ongoing PFS of > 52 months. Although immature, our data suggest a further survival benefit of adding ramucirumab to afatinib in patients with uncommon mutations. Future studies are recommended to assess the survival benefits of ramucirumab plus afatinib in patients with uncommon mutations.

Several other EGFR-TKI have been trialed in patients with EGFR-mutated metastatic NSCLC (such as dacomitinib and erlotinib) but with a high incidence and severity of adverse events [6]. In our study, dermatitis and paronychia were frequent among patients, although no significant difference was found between patients who were first treated with afatinib compared with patients treated with afatinib and ramucirumab. Diarrhea was frequent in both groups as well. Our results were in accordance with those reported by Paz-Ares et al., who also found that diarrhea (12.5%) was a frequent adverse event [28]. By contrast, other studies have reported hypertension and renal failure as the most frequent adverse events, which could not be managed with dose adjustments or supportive care [21].

While the present study provides novel findings regarding the benefit of afatinib plus ramucirumab for patients with EGFR-mutated metastatic NSCLC, we acknowledge that the study has certain limitations. The study’s findings are based on retrospective data collection, introducing reporting bias, and limiting the control of the outcomes reporting and definitions. In addition, the sample size of the included patients was relatively small, and the follow-up period was short to attain a measurable median PFS. We could not assess the impact of long-term response to afatinib monotherapy on the survival benefit of combination therapy due to the small sample size. Besides, the causal relationship between treatment regimen and survival benefit could not be established due to the lack of a control group.

## Conclusions

This real-world cohort study demonstrated that afatinib plus ramucirumab could improve the PFS of patients with EGFR-mutated metastatic NSCLC. The survival benefit was notable for the combination of afatinib and ramucirumab, at a predictable safety profile for both drugs. Additional data collected among a large population remains necessary to better address the advantages of afatinib plus ramucirumab and optimize their clinical applications. Although immature, our data suggest a further survival benefit of adding ramucirumab to afatinib in patients with uncommon mutations. Future studies are recommended to assess the survival benefits of ramucirumab plus afatinib in patients with uncommon mutations.

## Data Availability

The data used to support the findings of this study are included within the article.
